# A Phase II Pilot Trial to Evaluate *CoBaT-Y017* Safety and Efficacy against Uncomplicated Falciparum Malaria versus Artemether-Lumefantrine in Benin Subjects

**DOI:** 10.1155/2020/8715021

**Published:** 2020-02-17

**Authors:** Adrien N. Noudjiegbe, Femi N. Alikekere, Henri Tchehouenou, Yéman Langa, Daniel S. Ota, Jean-Eudes Degbelo, Aurel C. E. Allabi

**Affiliations:** ^1^Faculty of Health Sciences, Laboratory of Pharmacology and Toxicology, University of Abomey-Calavi, Cotonou, Benin; ^2^Beninese Center of Scientific Research and Innovation, National Laboratory of Narcotic and Toxicology, Cotonou, Benin; ^3^Teaching Hospital of Abomey-Calavi/ Sô-Ava, Laboratory of Biomedical Analysis, Cotonou, Benin; ^4^Service of Medicine, Teaching Hospital of Abomey-Calavi/ Sô-Ava, Cotonou, Benin

## Abstract

**Background:**

Considering the promising results of Phase I clinical trials with herbal medicine *CoBaT-Y017*, a Phase II study was conducted with *Plasmodium falciparum* malaria-infected patients, for efficacy and safety evaluation of *CoBaT-Y017*, a Phase II study was conducted with *Plasmodium falciparum* malaria-infected patients, for efficacy and safety evaluation of *CoBaT-Y017* compared with Artemether-Lumefantrine used as a positive control.

**Methods:**

A single-blind randomized trial was conducted on 25 eligible males aged 18–40 years randomly assigned to two treatment groups: *CoBaT-Y017*, a Phase II study was conducted with *Plasmodium falciparum* malaria-infected patients, for efficacy and safety evaluation of *CoBaT-Y017*, a Phase II study was conducted with *Plasmodium falciparum* malaria-infected patients, for efficacy and safety evaluation of *CoBaT-Y017* compared with Artemether-Lumefantrine used as a positive control. *Methods*. A single-blind randomized trial was conducted on 25 eligible males aged 18–40 years randomly assigned to two treatment groups: *CoBaT-Y017* or Artemether-Lumefantrine. The first group received 35 ml of *CoBaT-Y017* in 1.5 L mineral water administered daily for four consecutive days; the second group received oral Artemether-Lumefantrine, using WHO-recommended therapeutic dose regimens. For both drugs, efficacy for parasite clearance and safety were evaluated clinically, haematologically, and biochemically on days 1–4, 7, 14, 21, and 28. Clinical- and laboratory-adverse events (AEs) were recorded until day 28.

**Results:**

13 and 12 patients were randomized into *CoBaT-Y017*, a Phase II study was conducted with *Plasmodium falciparum* malaria-infected patients, for efficacy and safety evaluation of *CoBaT-Y017*, a Phase II study was conducted with *Plasmodium falciparum* malaria-infected patients, for efficacy and safety evaluation of *CoBaT-Y017* compared with Artemether-Lumefantrine used as a positive control. *Methods*. A single-blind randomized trial was conducted on 25 eligible males aged 18–40 years randomly assigned to two treatment groups: *CoBaT-Y017* or Artemether-Lumefantrine. The first group received 35 ml of *CoBaT-Y017* in 1.5 L mineral water administered daily for four consecutive days; the second group received oral Artemether-Lumefantrine, using WHO-recommended therapeutic dose regimens. For both drugs, efficacy for parasite clearance and safety were evaluated clinically, haematologically, and biochemically on days 1–4, 7, 14, 21, and 28. Clinical- and laboratory-adverse events (AEs) were recorded until day 28. *Results*. 13 and 12 patients were randomized into *CoBaT-Y017* arm and Artemether-Lumefantrine arm, respectively. In all patients, parasitaemia was adequately neutralized with *CoBaT-Y017* group patients' parasite clearance lagging slightly behind that of Artemether-Lumefantrine's group, but without a statistically significant difference (HR = 1.08, 95% CI 0.47–2.51, *P*=0.85). Physical and laboratory examinations did not show any significant changes in vital signs, biochemical, and haematological parameters. In the Artemether-Lumefantrine arm, 100% (12/12) of patients experienced, at least, one adverse event versus 61.5% (8/13) in the *CoBaT-Y017*, a Phase II study was conducted with *Plasmodium falciparum* malaria-infected patients, for efficacy and safety evaluation of *CoBaT-Y017* compared with Artemether-Lumefantrine used as a positive control. *Methods*. A single-blind randomized trial was conducted on 25 eligible males aged 18–40 years randomly assigned to two treatment groups: *CoBaT-Y017* or Artemether-Lumefantrine. The first group received 35 ml of *CoBaT-Y017* in 1.5 L mineral water administered daily for four consecutive days; the second group received oral Artemether-Lumefantrine, using WHO-recommended therapeutic dose regimens. For both drugs, efficacy for parasite clearance and safety were evaluated clinically, haematologically, and biochemically on days 1–4, 7, 14, 21, and 28. Clinical- and laboratory-adverse events (AEs) were recorded until day 28. *Results*. 13 and 12 patients were randomized into *CoBaT-Y017* arm and Artemether-Lumefantrine arm, respectively. In all patients, parasitaemia was adequately neutralized with *CoBaT-Y017* group patients' parasite clearance lagging slightly behind that of Artemether-Lumefantrine's group, but without a statistically significant difference (HR = 1.08, 95% CI 0.47–2.51, *P*=0.85). Physical and laboratory examinations did not show any significant changes in vital signs, biochemical, and haematological parameters. In the Artemether-Lumefantrine arm, 100% (12/12) of patients experienced, at least, one adverse event versus 61.5% (8/13) in the *CoBaT-Y017* arm.

**Conclusion:**

*CoBaT-Y017*, a Phase II study was conducted with *Plasmodium falciparum* malaria-infected patients, for efficacy and safety evaluation of *CoBaT-Y017* compared with Artemether-Lumefantrine used as a positive control. *Methods*. A single-blind randomized trial was conducted on 25 eligible males aged 18–40 years randomly assigned to two treatment groups: *CoBaT-Y017* or Artemether-Lumefantrine. The first group received 35 ml of *CoBaT-Y017* in 1.5 L mineral water administered daily for four consecutive days; the second group received oral Artemether-Lumefantrine, using WHO-recommended therapeutic dose regimens. For both drugs, efficacy for parasite clearance and safety were evaluated clinically, haematologically, and biochemically on days 1–4, 7, 14, 21, and 28. Clinical- and laboratory-adverse events (AEs) were recorded until day 28. *Results*. 13 and 12 patients were randomized into *CoBaT-Y017* arm and Artemether-Lumefantrine arm, respectively. In all patients, parasitaemia was adequately neutralized with *CoBaT-Y017* group patients' parasite clearance lagging slightly behind that of Artemether-Lumefantrine's group, but without a statistically significant difference (HR = 1.08, 95% CI 0.47–2.51, *P*=0.85). Physical and laboratory examinations did not show any significant changes in vital signs, biochemical, and haematological parameters. In the Artemether-Lumefantrine arm, 100% (12/12) of patients experienced, at least, one adverse event versus 61.5% (8/13) in the *CoBaT-Y017* arm. *Conclusion*. *CoBaT-Y017* exhibited similar antimalarial efficacy against *P. falciparum* to that of Artemether-Lumefantrine, with good tolerability and safety.*P. falciparum*

## 1. Introduction

The majority of antimalarial drugs have been derived from medicinal plants or made of chemical structures modelled on plant lead compounds.

Lessons from the past prove that progress towards “Malaria Elimination” entails both a judicious stewardship of existing treatments and the development of a steady stream of new drugs [1, 2]. *Plasmodium* parasite's resistance against earlier antimalarials is now widespread. Moreover, artemisinins, currently the most potent and fast-acting antimalarial [3], are associated with high recrudescence rates when used as monotherapy and must be combined with other drugs. Worryingly, artemisinin resistance is now being seen in the Greater Mekong subregion (southeast Asia) [4, 5]. This emergence of resistance to artemisinin and increased rate of treatment failures with artemisinin combination therapies [6, 7] highlight the need for new antimalarial drugs development not only from chemical synthesis but also from medicinal plants, to drive the elimination of malaria. Much research is made on traditional herbal medicines in Africa, but their clinical studies are scarce. Out of more than 1200 plant species reportedly used for the treatment of malaria, only 13 have undergone clinical trials, although hundreds have been tested in laboratory [8]. Furthermore, there are many herbal remedies available on the market without any proof of their safety or efficacy. However, to further widen their forum of acceptance, clinical trials of these phytomedicines should be performed. Considering that we are in resource-limited setting, a “reverse pharmacology” approach was used [9], to reduce the cost of drug development. The first step was to select a remedy for development, through a survey screening out the most used phytomedicine in Benin for uncomplicated malaria self-treatment. *CoBaT-Y017* was ranked as the leading antimalarial phytomedicine without marketing authorization. The second step was a preclinical study evaluating *CoBaT-Y017* acute and subacute toxicity. Satisfactorily results obtained led to Phase I study to establish the safety and tolerability of *CoBaT-Y017* and providing some indications on the antimalarial efficacy of the drug [10].

The aim of this study, as the third step of our “reverse pharmacology” approach, was a randomized controlled trial to compare the *CoBaT-Y017* efficacy and safety with the standard first-line treatment, AL.

## 2. Materials and Methods

### 2.1. Description of the Herbal Drug

*CoBaT-Y017* is an herbal medicinal product used by the population for uncomplicated malaria self-treatment. It is presented in the form of coffee-brown syrup accommodated in 70 mL bottles in a package including a measuring cup of 10 mL, manufactured by COPHARBIOTECH Ltd., a Beninese local company which develops some pharmaceutical products of category 2 according to the WHO phytomedicines classification. *CoBaT-Y017* has been formulated from two herbals mixture: *Mentha piperita* and *Cinnamomum zeylanicum*.

### 2.2. Study Design and Areas

The study was consisted in a Phase II randomized, single-blind clinical trial in patients with uncomplicated malaria to evaluate *CoBaT-Y017* efficacy and safety versus *AL*. It was conducted between March and September, covering the period June–August, the peak transmission season. All the activities were performed at the internal medicine department of the Teaching hospital, Abomey-Calavi/Sô-Ava.

### 2.3. Inclusion Criteria

The following inclusion criteria were adopted: male gender; aged between 18 and 40; currently infected only by *P. falciparum* detected by microscopy (asexual-stage parasite count, 300 to 110,000 parasites per microliter of whole blood, monoinfection); have axillary temperature higher than 37.5°C or history of fever during the past 24 h; having consumed no antimalarial within the previous 14 days; been treated with any medical or herbal drug within the previous 7 days; being able to swallow oral medication; have no chronic pathology; have no gastrointestinal intolerance to oral medication, including nausea, vomiting, and/or diarrhea; have no allergy to *CoBaT-Y017* and *AL*; have not undergone any substance abuse in the past 30 days; have not consumed any alcohol in the past week; being able and willing to comply with the protocol and visit the schedule of the study for its entire duration and sign an informed consent form.

### 2.4. Exclusion Criteria

The following exclusion criteria were adopted: clinical or laboratory signs of severe malaria according to the WHO definition during the study [11]; severe vomiting; having an evidence of significant clinical abnormalities detected by a physician after fulfilling the inclusion criteria; any kind of medical or herbal treatment intake during the study without the investigator' prior knowledge and consent; alcohol intake during the study.

### 2.5. Participants and Enrolment

On admission, patients were fully examined; blood samples were taken for full blood-cell count and routine blood biochemistry, confirmed of malaria by microscopy. Those patients who fulfilled the inclusion criteria were enrolled. The enrolled volunteers were randomized into one of two treatment arms. The investigator physicians and laboratory staff did not know the medical treatment administered to the patients, while the patients, the randomizer, and the nurse in charge of administering the drugs knew the exact treatment.

### 2.6. Treatments and Follow-Up

The patients of both study arms were orally treated.

For the *CoBaT-Y017* arm, 35 mL of *CoBaT-Y017* diluted in 1.5 L of mineral water was administered each day for 4 consecutive days.

For the *AL* arm, patients were treated with oral *AL*, Bimalaril®80/480 (tablets), using the WHO-recommended therapeutic dose regimens [11].

The study nurse supervised all the treatments and monitored the participants for 30 min for adverse reactions or vomiting following intake. Patients with fever ≥38.5°C were treated with paracetamol.

All participants were hospitalized during the four-day treatment and the fifth day to ensure strict monitoring. After five days in hospital, patients were medically checked at days 7, 14, 21, and 28, to ensure full recovery without complications while recording any adverse event. At each medically check, a clinical examination was performed, a parasitological evaluation according to the WHO protocol [12] was conducted, and blood samples were collected to assess haematological and biochemical parameters.

### 2.7. Efficacy Assessments

Thick and thin blood smears were performed before treatment (day 1) and days 2 to 5, 7, 14, 21, and 28 for both groups. Slides, using Giemsa stain, were examined through a microscope to determine parasite species and density according to the WHO protocol [12].

Parasite density (per *µ*l) was calculated assuming a white blood cell count of 8000/*µ*l. All slides underwent independent counts by two qualified microscopists, and the average of the two counts was taken as the parasite density. Parasite counts with discordant results (differences in species, presence of parasites, or parasite density >50%) were re-read by a third microscopist, and parasite densities were calculated by averaging the two closest counts. Density was calculated using the following formula:

(1) $$\begin{array}{c} \text{parasite density}\left(\frac{\text{parasites}}{\mu \text{L}}\right)=\frac{\text{number of parasites}\times 8000}{\text{number of WBCs}}. \end{array}$$

When the examination of 100 microscope fields on a specific thick-film did not show the presence of asexual forms of *P. falciparum*, the blood slide was reported negative.

The efficacy assessment was performed using Kaplan–Meier estimator applied to the parasite clearance time, fever clearance time, and cure rate up to day 28. Parasite clearance time relates to the evolution of the proportion of positive blood smears over time and cure rate corresponds to the evolution of the parasite density over time.

### 2.8. Safety and Tolerability Assessments

At screening, all patients underwent a complete physical examination, monitoring of vital signs, and routine laboratory testing. Blood samples were analyzed for potential haematological abnormalities including the number of white blood cells, neutrophils, lymphocytes, platelets, red blood cells, and ratios of haemoglobin and haematocrit. Hepatic function and renal function tests were conducted by assessment of blood levels of aspartate transaminase (AST) and alanine transaminase (ALT), creatinine, and urea. All symptoms and adverse events were recorded. Incidence of all adverse events was scored from 1 to 5 (1 = mild, 2 = moderate, 3 = severe, 4 = very severe, and 5 = Death related to AE), according to the Common Terminology Criteria for Adverse Events (CTCAEs) [13]. Adverse event frequencies were calculated.

### 2.9. Statistical Analysis

The sample size was calculated by determining the minimum size required for a 60% efficacy of *CoBaT-Y017* for a noninferiority study. Assuming that the risk of erroneously rejecting an effective substance is limited to *β* ≤ 0.05, the size NA of one of the two arms of Phase II satisfies the relation: NA size ≥ ln 0.05/Ln (1-p), NA ≥ 6. Consequently by doubling the size, two cohorts (*n* = 12 or 13) were recruited.

Treatment efficacy was determined with the calculations of the proportion of positive thick blood film and the parasite density variation over time (follow-up days) in each treatment group using parasitaemia determined by microscopy. Graphical displays of the Kaplan–Meier estimates for parasite clearance time and fever clearance time were generated. Chi-squared test or Fisher's exact tests were used for comparing differences in categorical data. A *P* value ≤ 0.05 was considered as significant.

### 2.10. Ethical Approval

This study was approved by the Research Ethics Committee of Applied Biomedical Sciences Institute (CER-ISBA) of Cotonou with the reference no. N103 du 09/01/17. The approval was renewed by the Ethics Committee on August 20, 2018. The study was conducted in accordance with the Declaration of Helsinki principles the International Committee of Harmonization Good Clinical Practice Guidelines (http://www.tga.gov.au/docs/pdf/euguide/ich/ich13595.pdf). All samples were coded with an ID number. All participants gave written informed consent and had the choice to withdraw from the study at any time.

## 3. Results

### 3.1. Study Patients and Baseline Parameters

**Figure 1:**
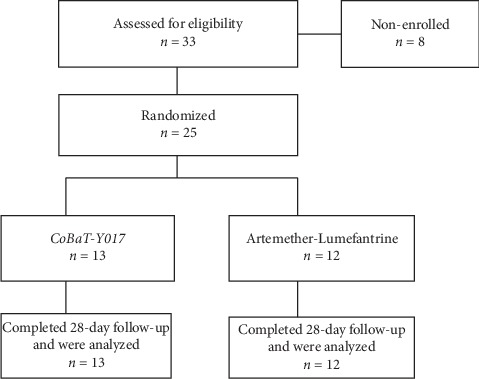
Participant distribution.

Among 33 males screened as potential study volunteers, 25 were deemed eligible and finally enrolled in two treatment arms (Figure 1). Reasons for exclusion of the eight rejected volunteers included the following: declined to participate (*n* = 2) and herbal or traditional medicine product intake within the last 7 days (*n* = 4) and hyperparasitaemia (*n* = 2). The median age was 26 years (range 18–40 years), and the median weight was 60 kg. Patients in both treatment groups had normal blood pressure at baseline with mean systolic arterial blood pressures of 11.61 ± 1.32 in the *CoBaT-Y017* group and 10.75 ± 0.68 in the *AL* group; the mean diastolic pressures were 7.54 ± 1.27 in the *CoBaT-Y017* group and 6.75 ± 0.62 in the *AL* group, respectively. All patients were feverish at the start of treatment with an average axillary temperature of 38.84 ± 1.06 and 38.5 ± 0.90 in the *CoBaT-Y017* group and *AL* group, respectively.

### 3.2. Evolution of the Proportion of the Thick Blood Film Positive and Parasite Density over Time (Days of Follow-Up) in Each Treatment Group

53.8% thick blood films for patients treated with *CoBaT-Y017* became negative 24 hours after administration versus 25% of those for *AL* patients. After 48 hours following the administration, 76.1% thick blood films were negative for the *CoBaT-Y017* arm versus 66.7% in the *AL* group. However, 72 hours after administration (day 3) 100% of the thick blood films were negative for the *AL* group against 84.6% for the *CoBaT-Y017* group. Two patients (15.4% of treatment group) diagnosed at day 3 as coinfection with *Salmonella* sp. had their thick blood film positive. Upon co-treatment of the salmonellosia (15.4%), those two patients had their parasitaemia canceled on day 5, while it was already cleared in all other participants for both arms at day 3. The decline of the positive thick blood films reflected the reduction of the parasite density in the treatment arms. The decrease of parasite density is rendered by the decreasing number of positive thick blood films (Table 1).

### 3.3. Parasite Clearance

**Figure 2:**
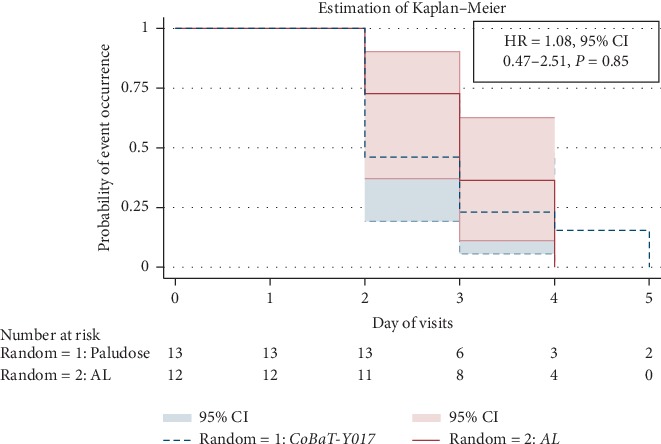
Parasitic clearance of *CoBaT-Y017* versus *AL*.

In all patients, parasitaemia cleared no later than day 5. Patients who received *CoBaT-Y017* had a slightly longer delay in parasite clearance (delayed parasite clearance) compared with those of the *AL* group, without a statistically significant difference (HR = 1.08, 95% CI 0.47–2.51, *P*=0.85). The parasite clearance in the two treatment arms is statistically best illustrated by the Kaplan–Meier estimator (Figure 2). In summary, there is virtually no difference between the antimalarial efficacy of *CoBaT-Y017* and that of *AL*.

### 3.4. Fever Clearance

**Figure 3:**
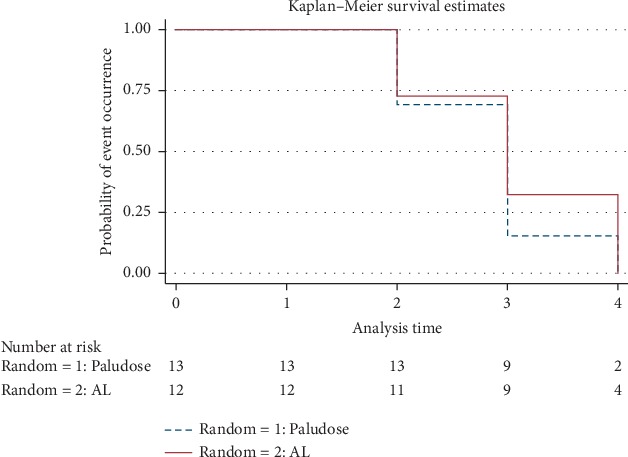
Delay of fever regression in both arms of the treatment.

Fever clearance in both treatment arms was illustrated by the Kaplan–Meier estimator (Figure 3).

### 3.5. Comparative Effects of *CoBaT-Y017* and *AL* on Haematological Parameters

The mean value of haemoglobin, red blood cell, and platelet remained almost constant before and after treatment in both arms of study (*P* > 0.05). However, a slight haemoglobin increase was noted in the *AL* arm at D28, but this was not followed up due to the termination of the study. As for the mean absolute value of leukocytes, a nonsignificant increase was observed on D3, D14, and D21 in *CoBaT-Y017* patients, while in the *AL* patients, this value remained quasi constant before and after treatment (Table 2).

The transaminase mean values in patients of both study arms at baseline (D1) were within normal limits. our study subjects having not experienced any liver dysfunction prior to treatment. However. the baseline values of AST and ALT transaminases of patients randomized in *CoBaT-Y017* arm were higher than the baseline values of the patients randomized in *AL* arm without a significant difference (*P*=0.30 for AST and *P*=0.38 for ALT). After treatment. no statistically significant changes in these values were observed in both study arms during the follow-up days. Likewise. the values of these hepatic parameters remained within the normal limits for both study arms (reference range 12–42 IU/L and 10–48 IU/L for AST and ALT. respectively) until day 28. *CoBaT-Y017* like *AL* did not alter the patients hepatic's functions (Table 3).

### 3.6. Comparative Effects of *CoBaT-Y017* and AL on Renal Parameters

The creatinine and urea mean values at baseline (D1) were within normal limits; patients of both arms had no renal dysfunction. In relation to creatinine. a nonsignificant increase was observed on D4 (*P*=0.32) with *CoBaT-Y017* patients when compared with those of *AL*. Besides this. no statistically significant changes (*P* > 0.05) were observed during the follow-up time. The mean values of urea remained almost constant before and after treatment in both arms of the study (*P* > 0.05). The values of the renal parameters' remained within the normal limits in both study arms; neither *CoBaT-Y017* nor *AL* altered the renal function of the patients (Table 4).

### 3.7. Adverse Events

In the *AL* arm. 100% (12/12) of patients experienced. at least. one adverse event. while in the *CoBaT-Y017* arm. only 61.5% (8/13) of patients have experienced adverse events. Headaches were the most reported adverse events in both treatment arms. with 66.7% and 30.8% for the *AL* and *CoBaT-Y017*. respectively; the second most frequent adverse event reported by 41.7% of patients only in the *AL* group was fatigue. Similarly. insomnia 8.3% (1/12). abdominal pain 8.3% (1/12). stiffness 16.7% (2/12). and rash 8.3% (1/12) were reported only in the *AL* arm. Adverse events reported only in the *CoBaT-Y017* arm include increased appetite 15.4% (2/13). vomiting 15.4% (2/13). and nausea 15.4% (2/13) (Table 4).

Spontaneous regression of adverse events was observed in the *CoBaT-Y017* arm at D4. whereas persistence of the same was observed in some patients (8.3%) in the *AL* arm until D7.

According to the CTCAE. all recorded adverse events recorded were categorized as grade 1 except headache reported as categorized grade 2 in both arms. No serious adverse event was reported in either treatment arm.

## 4. Discussion

According to information on hand. very few studies have been conducted on the validation of phytomedicines used for uncomplicated malaria [8]. While historically. the majority of antimalarial drugs have been derived from medicinal plants or made of chemical structures modelled on plants lead-compounds.

The aim of this study was to determine. on as a few subjects as possible. whether or not the *CoBaT-Y017* can be considered active and therefore be retained. More than one subject responded to this treatment. All thirteen patients enrolled in *CoBaT-Y017* arm were completely cured after four days of treatment.

Our investigation product. herbal medicine. was compared with a well-established first-line treatment recommended by the National Program of Malaria Control in Benin and by the WHO allowing to determine *CoBaT-Y017* useful in the therapeutic arsenal. This allowed us to estimate the response rate observed with a good level of precision.

The results showed a significant parasitaemia decrease in patients treated with *CoBaT-Y017* and *AL* with adequate clinical parasitological responses (APCRs) at day 28. No significant difference was observed for parasite clearance and rate of parasite regression between *CoBaT-Y017* and *AL* groups (*P*=0.85) (Figure 3).

Similar results were obtained in another study. 87.9% APCR for PR 259 CT1. an antimalarial phytomedicine versus 96.9% APCR for Artesunate-Amodiaquine combination [14]. Our results were also similar to that observed with *Argemone mexicana* decoction. an antimalarial phytomedicine for which a second-line treatment was not required for 89% of patients versus 95% of patients on AS-AQ [15]. For the present study. the second-line treatment for malaria was not required for any patient in both groups.

This finding supports the clinical information on the most used antimalarial phytomedicines which was ranked *CoBaT-Y017* as the leading antimalarial phytomedicine used in Benin for uncomplicated malaria self-treatment and that of the Phase I study [10].

It should be pointed out that the phytochemical screening of the batch of *CoBaT-Y017* that we used revealed the presence of six classes of chemical compounds including. alkaloids. flavonoids. and terpenes. Phenochemicals such as alkaloids. terpene. and its derivatives have been proven to be involved in the anti-*Plasmodium* activity of many plants [9. 15. 16].

After 28 days of follow-up. we did not observe any significant differences between the mean values of the different biological parameters in the two treatment groups.

For haemogram parameters. the mean value of haemoglobin. red blood cell. and platelet remained almost constant before and after treatment in both arms of the study (*P* > 0.05). Moreover. for the mean absolute value of leukocytes. a nonsignificant increase was observed on D3. D14. and D21 in *CoBaT-Y017* patients. which probably conferred it. some immunostimulatory properties.

With regard to renal tolerance. serum creatinine and urea were maintained within the normal limits in both study arms. indicating that *CoBaT-Y017* did not alter the renal function of the patients. Good hepatic tolerance was also seen in both treatment groups. *CoBaT-Y017* did not alter the patients' hepatic's functions. This hepatic profile observed with *CoBaT-Y017* is similar to what was observed with the extract of *Nauclea pobiguinii* [17]. an antimalarial herbal medicine. *CoBaT-Y017* hepatic tolerance was better compared with that of ferroquine. a candidate antimalarial currently undergoing clinical trials. for which transient elevated transaminase levels out of the normal limit were observed [18]. Compared with others studies. *CoBaT-Y017* liver tolerability was better than that of the combination AS-AQ for which severe increased of AST and ALT was observed in some patients [19]. and that of dihydroartemisinine-piperquin for which transaminases in some patients' day-7 levels were ≥50%. higher than the upper limit of normal [20].

Also. this hepatic safety of *CoBaT-Y017* seems safer compared with that of the extract of *Elaeis guineens* JACQ [21]. another antimalarial herbal medicine on the Benin market. For the latter. diarrhea was the most frequent adverse event (93%). followed by asthenia (20%). These adverse events have not been reported under *CoBaT-Y017* treatment. Compared with the clinical safety of PR 259 CT1 [17]. *CoBaT-Y017* appears to have fewer adverse events.

More adverse events have been reported in the *AL* combination compared with *CoBaT-Y017*. On D2 of the treatment. 100% of patients treated with *AL* had at least one adverse event compared with 46.2% of patients treated with *CoBaT-Y017*. In the latter. no adverse event was recorded after the end of treatment (D4). while some adverse events were still recorded with some *AL* group patients (8.3%) until D7. Headache was reported as the most frequent adverse event in both study arms but with a higher proportion in the *AL* group 66.7% for *AL* versus 30.8% for *CoBaT-Y017*. Fatigue was only recorded in the *AL* arm. which comes after the headaches in terms of frequencies. Similar data were obtained in another comparative study in which headache accounted for a higher proportion of adverse events followed by fatigue in patients treated with *AL* [22].

The main interest of this work was that we started from the realities of field. remedy used informally. and without marketing authorization. to appreciate its safety and tolerability [11] and then evaluate its efficacy in uncomplicated falciparum malaria treatment.

The efficacy of *CoBaT-Y017* would be due to the synergic effect of its components. Further studies are planned to identify active compounds. which could be used as markers and demonstrate the mechanism(s) of action. That should be the next step of our simplified approach called “reverse pharmacology” [9] to develop more quickly and cheaply an antimalarial phytomedicine.

We can conclude that *CoBaT-Y017* has a proven antimalarial efficacy and has good haematological. hepatic. and renal tolerance. The benefit exhibited by *CoBaT-Y017* at the end of a 28 day follow-up outweighs by far the risk it presents. when compared with artemisinin-based combination therapies or other antimalarial phytomedicines available on the Benin market.

## Figures and Tables

**Table 1:** Evolution of the positive thick blood film and the parasite density in the two arms of study at different days of visit.

Biological characteristics	Day	*AL* group	*CoBaT-Y017* group	*P* value^*∗*^
		Proportion or mean (± std)	Proportion or mean ( ± std)	
Positive thick blood film	D1	100%	100%	
	D2	75%	46.2%	0.14
	D3	33.3%	23.1%	0.67
	D4^*∗∗*^	—	15.4%	
	D5	—	—	
	D7	—	—	
	D14	—	—	
	D21	—	—	
	D28	—	—	

Parasite density	D1	14482.4 (±7888)	17832.4 (±28442)	0.77
	D2	6375.3 (±17689)	4335.5 (±9385)	0.72
	D3	172.6 (±352)	1785.2 (±4846.4)	0.26
	D4^*∗∗*^		367.3 ((±1131)	—
	D5	—	—	
	D7	—	—	
	D14	—	—	
	D21	—	—	
	D28	—	—	

^*∗*^Fisher's exact nonparametric test; ^*∗∗*^D4-positive parasitaemia was only observed in the *CoBaT-Y017* group; —: negative thick blood film.

**Table 2:** Variation in haematologic parameters of patients in both treatment arms.

Parameters	Treatment arms	D1 (baseline)	D2	D3	D4	D7	D14	D21	D28
Hb (rate) (×10^1^)	*CoBaT-Y017*	13.7 ± 1.9	13.5 ± 1.8	13.3 ± 2.1	13.3 ± 1.8	13.2 ± 1.9	12.9 ± 1.5	13.2 ± 1.7	13.4 ± 1.6
	*AL*	13.5 ± 1.7	13.5 ± 2.1	12.92 ± 1.9	12.54 ± 1.2	12.66 ± 1.5	13.25 ± 1.1	13.58 ± 1.1	**16.0** **±** **6.7**

RC (×10^9^)	*CoBaT-Y017*	5.23 ± 1.4	4.69 ± 0.6	4.76 ± 0.8	4.84 ± 0.7	4.61 ± 0.8	4.53 ± 0.8	4.69 ± 0.8	4.61 ± 0.7
	*AL*	5 ± 0.6	4.75 ± 0.9	4.33 ± 0.8	4.63 ± 0.7	4.75 ± 0.8	4.83 ± 0.7	5 ± 0.6	5.16 ± 0.8

WC (×10^3^)	*CoBaT-Y017*	5.4 ± 2.1	5.4 ± 2.8	**9.3** **±** **4.8**	4.8 ± 1.3	6.6 ± 2.8	**9.5** **±** **4.1**	**9.15** **±** **4.1**	4.2 ± 1.9
	*AL*	4.6 ± 1.2	4.9 ± 1.6	**4.5** **±** **2.02**	5.1 ± 1.9	5.4 ± 1.5	**5.5** **±** **1.5**	**4.5** **±** **1.1**	4.3 ± 1.7

Plat. (×10^3^)	*CoBaT-Y017*	0.2 ± 0.06	0.17 ± 0.09	0.2 ± 0.08	0.2 ± 0.08	0.2 ± 0.08	0.3 ± 0.2	0.3 ± 0.08	0.2 ± 0.08
	*AL*	0.2 ± 0.1	0.2 ± 0.09	0.2 ± 0.09	0.2 ± 0.12	0.3 ± 0.2	0.4 ± 0.1	0.3 ± 0.06	0.2 ± 0.04

RC: red blood cells; WC: white cells; Plat: platelets; Hb: haemoglobin. Comparative effects of *CoBaT-Y017* and AL on hepatic parameters.

**Table 3:** Variation of mean of AST and ALT values of patients at different follow-up days in the study arms.

Parameters	Treatment arms	D1 (baseline)	D4	D7	D14	D21	D28
AST	*CoBaT-Y017*	**25.66** **±** **18.29**	28.41 ± 15.46	28.20 ± 17.26	22.96 ± 14.89	19.07 ± 5.40	20.81 ± 7.95
	*AL*	**19.06** **±** **11.89**	19.09 ± 6.60	15.85 ± 5.78	22.26 ± 9.50	19.72 ± 9.53	15.88 ± 4.17

ALT	*CoBaT-Y017*	**26.94** **±** **18.89**	29.91 ± 17.22	34.25 ± 23.16	22.79 ± 9.30	24.64 ± 18.98	21.21 ± 10.61
	*AL*	**21.12** **±** **13.83**	19.85 ± 10.06	19.15 ± 6.72	22.69 ± 14.63	21.06 ± 12.85	19.49 ± 10.17

Values were expressed as mean ± SD.

**Table 4:** Variation of mean values of creatinine and urea at different follow-up days in the study arms.

Parameters	Treatment arms	D1 (baseline)	D4	D7	D14	D21	D28
Creatinine	*CoBaT-Y017*	11.50 ± 1.85	**17.73** **±** **22.33**	10.86 ± 1.80	11.40 ± 2.73	10.43 ± 1.39	11.08 ± 11.68
	*AL*	10.51 ± 3.7	**10.83** **±** **3.52**	12.37 ± 5.05	10.33 ± 1.71	10.46 ± 1.49	10.30 ± 1.77

Urea	*CoBaT-Y017*	0.14 ± 0.05	0.19 ± 0.21	0.15 ± 0.36	0.15 ± 0.07	0.15 ± 0.05	0.12 ± 0.03
	*AL*	0.15 ± 0.06	0.14 ± 0.04	0.18 ± 0.14	0.13 ± 0.03	0.15 ± 0.06	0.20 ± 0.20

Values are expressed as mean ± SD.

**Table 5:** Incidence of adverse events in both treatment arms.

(Adverse events (*n* (%) *E*)	*CoBaT-Y017*: *n* = 13	*AL*: *n* = 12
Increased appetite	2 (15.4 (%) 4)	(0 (%) 0)
Headache	4 (30.8 (%) 6)	8 (66.7 (%) 11)
Fatigue	0 (0 (%) 0)	5 (41.7 (%) 8)
Drowsiness	2 (15.4 (%) 3)	1 (8.3 (%) 1)
Vomiting	2 (15.4 (%) 2)	0 (0 (%) 0)
Nausea	2 (15.4 (%) 2)	0 (0 (%) 0)
Insomnia	0 (0 (%) 0)	1 (8.3 (%) 2)
Abdominal pain	0 (0 (%) 0)	1 (8.3 (%) 2)
Stiffness	0 (0 (%) 0)	2 (16.7 (%) 2)
Rash	0 (0 (%) 0)	1 (8.3 (%) 3)
No adverse events	5 (38.5%)	0 (0 (%) 0)

*n*: number; %: percent; *E*: episode.

## Data Availability

The datasets used and/or analyzed during the current study are available from the corresponding author on reasonable request.

## List of Abbreviations

ACPR: Adequate clinical and parasitological response
AE: Adverse event
AL: Artemether-Lumefantrine
ALT: Alanine transaminase
AST: Aspartate transaminase
AS-AQ: Artesunate-amodiaquine
CTCAE: Common Terminology Criteria for Adverse Events
D: Day
NA: Number of patients by the study arm
SD: Standard deviation.

## Additional Points

The limitations of this study are as follows: the nondetermination of adequate clinical and parasitological responses adjusted by PCR at day 28 and nonevaluation of cardiac safety.
